# The pan-PPAR agonist lanifibranor reduces development of lung fibrosis and attenuates cardiorespiratory manifestations in a transgenic mouse model of systemic sclerosis

**DOI:** 10.1186/s13075-021-02592-x

**Published:** 2021-09-06

**Authors:** Emma Derrett-Smith, Kristina E. N. Clark, Xu Shiwen, David J. Abraham, Rachel K. Hoyles, Olivier Lacombe, Pierre Broqua, Jean Louis Junien, Irena Konstantinova, Voon H. Ong, Christopher P. Denton

**Affiliations:** 1 Centre for Rheumatology and Connective Tissue Diseases, UCL Division of Medicine, Rowland Hill St., London, NW3 2PF UK; 2 Churchill Hospital, Oxford, OX3 7LE UK; 3 Inventiva, 21121 Daix, France

**Keywords:** Systemic sclerosis, Animal models, PPAR, TGFβ, Pulmonary hypertension, Pulmonary fibrosis

## Abstract

**Background:**

The TβRII∆k-fib transgenic (TG) mouse model of scleroderma replicates key fibrotic and vasculopathic complications of systemic sclerosis through fibroblast-directed upregulation of TGFβ signalling. We have examined peroxisome proliferator-activated receptor (PPAR) pathway perturbation in this model and explored the impact of the pan-PPAR agonist lanifibranor on the cardiorespiratory phenotype.

**Methods:**

PPAR pathway gene and protein expression differences from TG and WT sex-matched littermate mice were determined at baseline and following administration of one of two doses of lanifibranor (30 mg/kg or 100 mg/kg) or vehicle administered by daily oral gavage up to 4 weeks. The prevention of bleomycin-induced lung fibrosis and SU5416-induced pulmonary hypertension by lanifibranor was explored.

**Results:**

Gene expression data were consistent with the downregulation of the PPAR pathway in the TβRII∆k-fib mouse model. TG mice treated with high-dose lanifibranor demonstrated significant protection from lung fibrosis after bleomycin and from right ventricular hypertrophy following induction of pulmonary hypertension by SU5416, despite no significant change in right ventricular systolic pressure.

**Conclusions:**

In the TβRII∆k-fib mouse strain, treatment with 100 mg/kg lanifibranor reduces the development of lung fibrosis and right ventricular hypertrophy induced by bleomycin or SU5416, respectively. Reduced PPAR activity may contribute to the exaggerated fibroproliferative response to tissue injury in this transgenic model of scleroderma and its pulmonary complications.

## Background

Systemic sclerosis (scleroderma; SSc) is a fibrotic disease, where skin and internal organ fibrosis results from a pathological triad of vasculopathy, autoimmune inflammation and propensity to scarring. The clinical heterogeneity and biological complexity have resulted in the description of multiple and disparate pathophysiological aberrations, but alterations in transforming growth factor-β systems biology underlie many of the pro-fibrotic mechanisms and this cytokine is therefore the key pathological link in considering the underlying disease mechanism (reviewed in [1]). Leading causes of mortality in the current era commonly relate to cardiorespiratory complications of this disease [2].

The TβRII∆k-fib mouse model of systemic sclerosis expresses a kinase-deficient transforming growth factor β (TGFβ) type II receptor driven by a fibroblast-specific promoter-enhancer regulatory sequence subcloned from *Col1a2*, leads to ligand-dependent upregulation of TGFβ signalling in fibroblasts only, replicating key fibrotic and vasculopathic features of scleroderma. The phenotype and mechanism of this model have been published previously and are summarised in references [3–8]. In brief, the transgene stabilises endogenous wildtype receptor complexes leading to increased expression of the corresponding murine TβRII on fibroblasts that causes upregulated ligand-dependent TGFβ signalling in the skin and other connective tissues. This mouse strain develops constitutive skin fibrosis [3] and a structural vasculopathy [6] with vessel wall fibrosis. Approximately 25% of mice develop relatively mild spontaneous lung fibrosis [3] but more relevant to human SSc is the susceptibility to both pulmonary fibrosis in response to minor epithelial injury [4] and pulmonary endothelial cell injury (by SU5416) which leads to pulmonary hypertension and right ventricular strain [5, 7]. Administration of bleomycin leads to persistent and severe lung fibrosis when compared to unbuffered saline in transgenic (TG) mice or bleomycin in wildtype (WT) mice [4].

There is strong and emerging evidence for the role of peroxisome proliferator-activated receptors (PPARs) across all cardiovascular health and disease states including SSc. They serve as important regulators in many physiological processes that impact respiratory and cardiovascular function: modulation of the metabolism of carbohydrates, lipids and proteins; cellular proliferation; and differentiation and inflammation. Specifically, all receptors are implicated in the development of and, conversely, protection from pulmonary hypertension and particularly responses to endothelial stressors (reviewed in [9–11]). Each PPAR isoform has different effects on the vascular endothelium and smooth muscle layer, but PPAR β/δ is highly expressed and its activation is responsible for endothelial cell proliferation, NO release and inhibition of endothelial cell apoptosis [12]. There is functional interaction at multiple levels between PPAR and TGFβ signalling. Strong data have emerged regarding the positive role for pioglitazone, a PPARγ agonist in the treatment of pulmonary arterial hypertension and prevention of right ventricular failure in this condition [13].

The pan-peroxisome proliferator-activated receptor (PPAR) agonist lanifibranor has recently completed testing in a phase IIb clinical trial in non-alcoholic steatohepatitis, reaching both primary and key secondary endpoints successfully. A phase II trial in scleroderma did not reach the primary endpoint, but lanifibranor has previously been shown to ameliorate the development of scleroderma lung fibrosis and pulmonary hypertension in two mouse models [14]. This study investigates PPAR pathway perturbation in the TβRII∆k-fib transgenic mouse model, including baseline expression of PPAR, and the impact of PPAR stimulation on lung fibrosis and pulmonary hypertension in this animal model.

## Methods

### Generation of TβRIIΔk-fib–transgenic mice

The generation of TβRIIΔk-fib–transgenic mice has been described previously [3]. Each experiment was performed by comparing at least 6 mice for each condition and sex-matched littermate controls, and then, the experiments were performed in triplicate. Mice were aged 6–8 weeks in all the experiments except the haemodynamics experiments, in which, for technical reasons, the mice were aged 10–12 weeks. All measurements were made by observers blinded to treatment category or genotype. The mice were housed in a clean conventional colony, with access to food and water ad libitum. Strict adherence to institutional guidelines was practiced, and local ethics committee and Home Office approval were obtained prior to all animal procedures.

### Lanifibranor administration

Lanifibranor (3 mg/mL and 10 mg/mL) were formulated in 1% methylcellulose as ready-to-use suspensions and stored at 5 ± 3 °C during the study. One percent of methylcellulose was used as a vehicle and stored at 5 ± 3 °C during the study. Administration by oral gavage of a 200-uL drug or vehicle was commenced on day 1 of the experiment until sacrifice at day 21. Weights were recorded thrice weekly.

### Induction of lung fibrosis with bleomycin

Bleomycin sulphate (Sigma) was freshly dissolved in 0.9% NaCl. On day 3, mice were anaesthetized with inhaled 1.5% isoflurane and 200 µl bleomycin sulphate in 0.9% NaCl or 0.9% NaCl only was administered to the oropharynx with a micropipette to allow the mice to inhale this solution [15].

For this series of experiments, 12 comparator experimental groups were produced: 6 transgenic groups treated with 100 mg/kg daily lanifibranor, 30 mg/kg lanifibranor or vehicle only in which either bleomycin or unbuffered saline was used. These were annotated: TG-bleomycin 100, TG-bleomycin 30, TG-bleomycin vehicle, TG-saline 100, TG-saline 30 and TG-saline vehicle. There were a further 6 equivalent WT groups: WT-bleomycin 100, WT-bleomycin 30, WT-bleomycin vehicle, WT-saline 100, WT-saline 30 and WT-saline vehicle.

### Induction of pulmonary hypertension using SU5416

Reflecting its relevance as a model of organ-based complications of human systemic sclerosis, the TβRIIΔk-fib mice were given a single 50 mg/kg intraperitoneal injection of SU5416 (Sigma) in carboxymethylcellulose vehicle administered on day 3 to induce pulmonary endothelial apoptosis and subsequent endovascular proliferation leading to pulmonary hypertension. Previous data have shown that SU5416 administered in this way does not have any significant effect on wild-type animals of this strain, but TβRIIΔk-fib mice develop elevated pulmonary artery pressures and histological changes similar to human pulmonary hypertension, with endothelial proliferation causing vessel obliteration in around 20% of pulmonary arterioles [7].

### In vivo measurement of mean arterial blood pressure (MABP), right ventricular systolic pressure (RVSP), and RV hypertrophy

Hemodynamic measurements of RVSP and MABP were obtained in 12-week-old male TβRIIΔk-fib mice and wild-type (WT) littermate mice (20–25 g). The mice were anaesthetised with 1.5% isoflurane and placed in a supine position onto a heating blanket that was thermostatically controlled at 37 °C. First, the right jugular vein was isolated, and a Millar SPR-671NR mouse pressure catheter with a diameter of 1.4F was introduced and advanced into the right ventricle to determine RVSP. Next, mean arterial blood pressure was measured by isolating the left common carotid artery and introducing a pressure catheter. Both RVSP and mean arterial blood pressure measurements were recorded into a precalibrated PowerLab System (AD Instruments). The mice were sacrificed by isoflurane anaesthetic overdose, the lungs and heart were dissected, and the weights of the right and left ventricles were recorded.

### Fibroblast and aortic smooth muscle cell culture

#### Fibroblasts were grown from the lung or skin of neonatal and 6–8 weeks old

TβRIIΔk-fib mice and sex-matched wild-type littermates according to standard departmental protocols. Phase-contrast inverted microscopy confirmed the absence of epithelial cells and verified fibroblast morphology. Aortae were dissected, the adventitia stripped, and the vessel opened longitudinally. After collagenase digestion (1 mg/ml) for 10 min at 37 °C applied to the endothelial surface, the remaining smooth muscle cells were grown by explant culture in standard conditions. Immunostaining revealed > 99% α-SMA positivity at day 14. Experiments were performed at passages 3 to 4. RNA was extracted using the RNeasy Mini kit (Qiagen, UK).

### Histological analysis

Lung and cardiac tissue specimens were immersed in 10% formal saline or RNAlater (Qiagen). Formalin-fixed, paraffin-embedded sections were stained with haematoxylin and eosin (H&E) and picrosirius red according to standard protocols. For immunohistochemistry, the primary antibodies were PPAR α (dilution 1/200), PPAR δ (dilution 1/200) and PPAR γ (dilution 1/200) (all Abcam), α-smooth muscle actin (Sigma-Aldrich), CD31 (Abcam) and Ki-67 (Proteintech). PPAR α, PPAR δ and PPAR γ antibodies were all diluted to a concentration of 5 μg/ml. Concentration for CD31 antibody was 0.034 μg/ml, and concentration of Ki-67 was 0.12 μg/ml. Sections were viewed, and measurements quantified with either an Axioskop Mot Plus microscope using AxioVision software (Zeiss) or NanoZoomer S210 (Hamamatsu).

Following sacrifice on day 23, the whole right lung was dissected en bloc and instillation of PBS was performed before fixation in 10% formalin for 24 h. Samples were processed and embedded horizontally in composite blocks of 3 samples per block. Contiguous 3μm sections were stained using H&E according to standard protocols. Ashcroft score was performed on 5 fields using light microscopy at × 10 magnification. Two blinded observers independently scored each field from 0 (no fibrosis) to 8 (maximum fibrosis) [16]. Criteria for grading lung fibrosis were as follows: grade 0, normal lung; grade 1, minimal fibrous thickening of alveolar or bronchiolar walls; grade 3, moderate thickening of walls without obvious damage to lung architecture; grade 5, increased fibrosis with definite damage to lung structure and formation of fibrous bands or small fibrous masses; grade 7, severe distortion of the structure and large fibrous areas; and grade 8, total fibrous obliteration of fields. Results were collated for each mouse. Where scores differed by more than 2 points, these were resolved by consensus agreement.

### RNA quantitation and analysis

Total RNA was extracted, quantified, and subjected to quantitative reverse transcription-polymerase chain reaction analysis, as described previously [6]. The minimum 260:280 ratio was 1.9. RNA integrity values were > 8.8. The reference genes Sdha, Rpl13, ActB and Ubc were used to compute a normalisation factor [17].

Gene expression differences were quantified using MouseRef-8v1.1 expression BeadChips (Illumina, San Diego, CA) [18]. After normalisation (global; Bioconductor Lumi package, Seattle, WA), the genes were ranked according to differential expression. Data were expressed as pairwise analysis comparing the mean expression of two groups (*t* test), with the false discovery rate correction for multiple testing (number of tests/rank of *P* value). The PPAR pathway gene expression list obtained from http://software.broadinstitute.org/gsea/msigdb/cards/KEGG_PPAR_SIGNALING_PATHWAY was cross-referenced with each microarray. Forty-two Illumina microarray gene expression profiles were analysed. Within the GSEA cohort of genes, the number with significant differential expression or a trend to difference (*P* < 0.10) in the analysis was determined. A total list of 12,800 genes was tested, summarising 3 WT or littermate TG pairs for each of the test tissue or cell culture types.

### Biochemical analysis

The upper lobe of the left lung was snap-frozen in liquid nitrogen and stored at − 80 °C. Samples were weighed and a 30-mg sample was minced and subjected to collagen extraction using the cold Acid-Pepsin protocol followed by the collagen isolation and concentration protocol. A 40-uL sample was then analysed using the Sircol® assay according to manufacturer’s instructions and quantified by comparison to duplicate control bovine collagen samples of 0, 2.5, 5, 10 and 15μg collagen.

### Statistical analysis

Except where indicated otherwise, data are presented as the mean ± SEM of *n* observations. Statistical analysis was performed using Student’s *t* test or analysis of variance with post hoc correction for pairwise comparisons. *P* values < 0.05 were considered significant.

## Results

### PPAR gene expression is altered in whole skin, lung, and explanted cells of TβRII∆k-fib mice

**Fig. 1:**
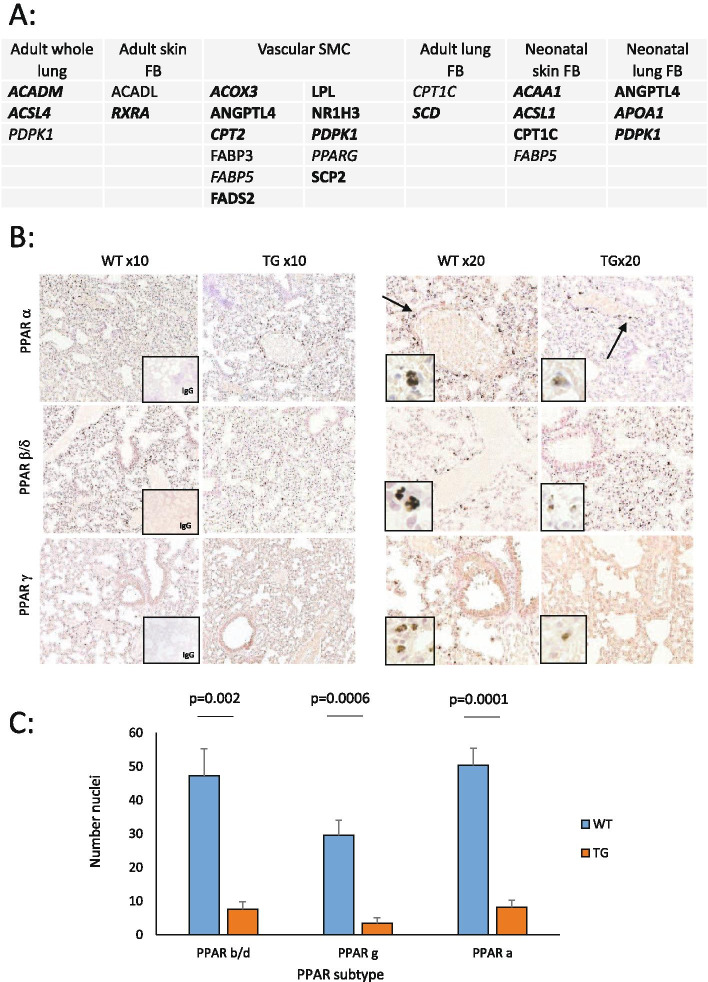
Dysregulated PPAR-associated gene and protein expression in TβRII∆k-fib mouse (TG) skin, lung, explanted fibroblasts and aortic smooth muscle cells. **A** Illumina gene array expression across tissues and explanted cells from TG compared to wildtype (WT) littermates. Bold- downregulated in TG; not bold- upregulated in TG; normal font- significant, *P* < 0.05; italicised font- trend only, *P* < 0.10; FB = fibroblast explant culture; aSMC = aortic smooth muscle cell explant culture, *n* = 3 all groups. **B** Representative immunohistochemical stains for PPAR subtypes in WT and TG, low power (× 10) and high power (× 20) including IgG control (*n* ≥ 3 each group). **C** Number of positive nuclei per high power field (HPF) in TG and WT immunohistochemical stains for each PPAR subtype

For individual substrate RNAs analysed, the number of genes were whole lung (*n* = 3), adult skin fibroblasts (*n* = 2), neonatal skin fibroblasts (*n* = 4), neonatal lung fibroblasts (*n* = 3) and adult vascular smooth muscle cells (*n* = 11). Overall, for the pathway genes within the GSEA cohort 42 of 69 annotated genes showed expression and were included in the Illumina microarray for these samples. Other genes were not present or did not show a significant signal for evaluation. Genes showing expression are summarised in Table A of Fig. 1, including those showing a trend of difference at the microarray level (italicised), or a significant difference (normal font, *P* < 0.05). Overall, 25 genes showed a trend of difference in at least one of the test substrates, and of these, the majority, 18 (72%) were downregulated in TG mice, identified in bold in Fig. 1A. For the smaller number of genes (*n* = 9) with a significant difference between WT and TG explant cells, 89% (*n* = 8) were reduced in the transgenic cells. Thus, for the evaluated genes where there was a difference in transgenic cells, this suggested overall a weak downregulation of the PPAR pathway. Also shown in Fig. 1, there were most differences for the aortic smooth muscle cells, and this is notable as the altered phenotype of these in culture is likely to reflect an altered in vivo environment, with elevated TGFβ activity in the vessel wall rather than intrinsic smooth muscle cell abnormalities since the SMC are not predicted to express the kinase-deficient TβRII transgene that is fibroblast-specific. This mirrors the findings of earlier experiments in this model [3].

Immunohistochemistry of PPAR isoform expression in TG and WT lung supported this finding, with nuclear expression of PPAR isoforms present in both WT and TG tissue. Higher expression was identified in proximity to vessel walls (arrows, Fig. 1B), particularly in WT animals and both numbers of nuclei stained positive, and strength of stain were lower in TG animals. More nuclear staining was detected with a PPARα-specific antibody compared to the antibodies against the other isoforms in these experiments. Representative sections are shown in Fig. 1B.

### Oro-pharyngeal bleomycin results in severe fibrosis in TβRII∆k-fib mice

**Fig. 2:**
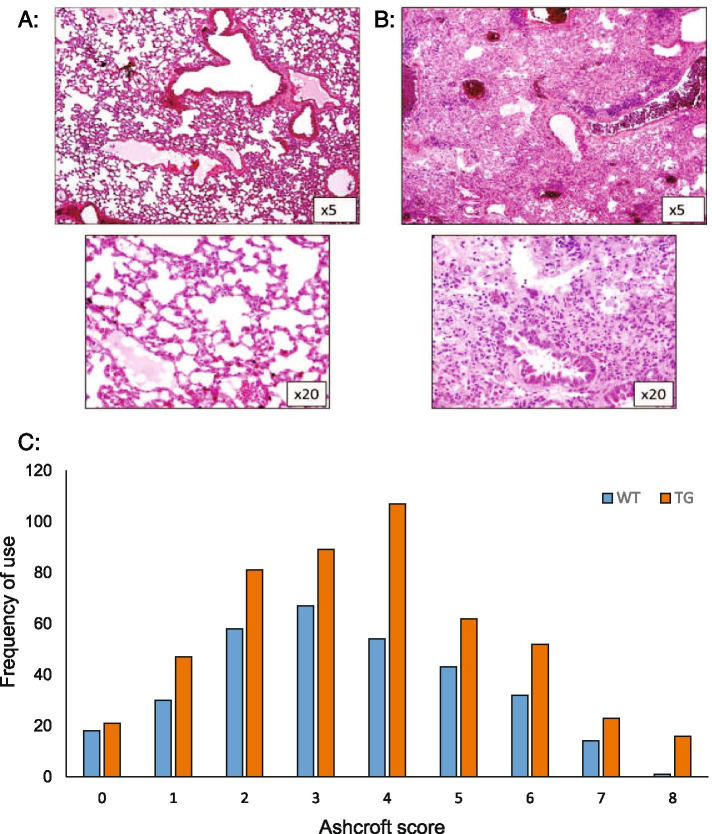
Oro-pharyngeal administration of bleomycin induces severe lung fibrosis in transgenic mice. Representative low (× 5) and high (× 20) power images of normal mouse lung (**A** Ashcroft score 0) and total fibrous obliteration of the lung following bleomycin administration in TG mice (**B** Ashcroft score 8). **C** Distribution of Ashcroft scores across all treatment groups for bleomycin experiments (*n* = 815 sections)

Oro-pharyngeal bleomycin administration to induce lung fibrosis had not been used in this model previously. We confirmed that this technique produced an equivalent severe degree of lung fibrosis in bleomycin-treated TG animals when administered vehicle compared with the previously used method of intra-tracheal bleomycin administration (representative images are shown in Fig. 2A and B). The Ashcroft score is routinely used to assess the degree of fibrosis in mouse lung tissue. Studies have proven difficult because fibrosis in the lung is heterogeneous, often with some area of the lung completely spared. This can lead to variability in scoring, and incomplete fibrosis so that the full spectrum of scores cannot be used. Therefore, in this study, multiple fields were examined so that the whole lung parenchyma was scored for each animal on each slide, and multiple cuts examined per sample. As a result, in this experiment, all scores between 0 and 8 were utilised, the modal score was 3. Full data are presented in Fig. 2C, including representative H&E sections comparing score 0 with score 8. These data show both that oro-pharyngeal administration of bleomycin has a similar effect to the previously reported technique of intra-tracheal administration [4] and that the scoring technique used was appropriate to differentiate the degree of fibrosis observed in this study.

### PPAR agonist lanifibranor attenuates bleomycin-induced lung fibrosis in TβRII∆k-fib mice

**Fig. 3:**
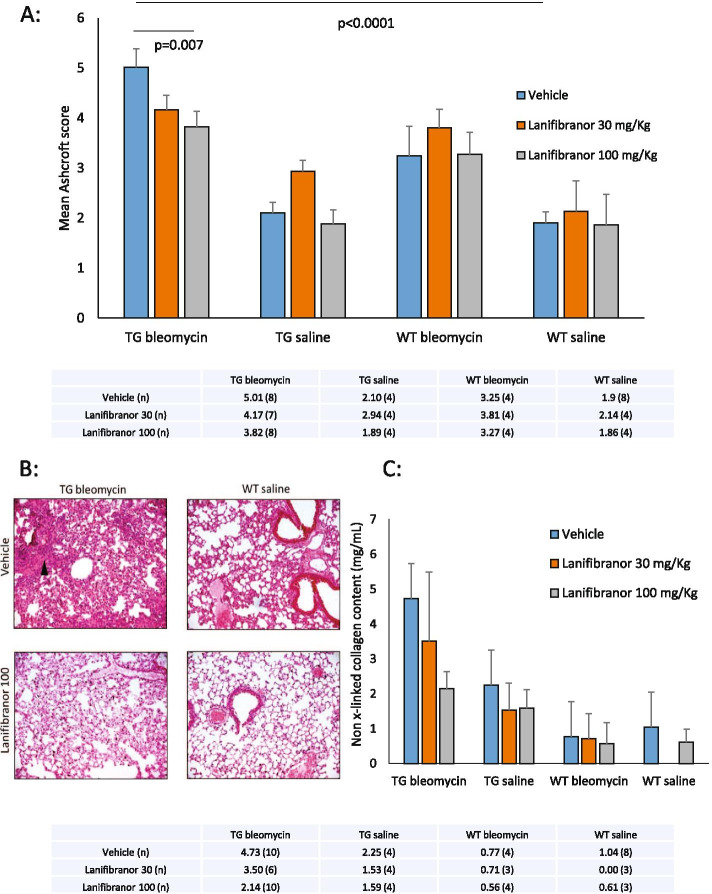
Prevention of bleomycin-induced lung fibrosis using PPAR agonist lanifibranor in TβRII∆k-fib mice. **A** Histogram showing mean Ashcroft score across 12 treatment groups; with detailed mean (*n*) data table below; *n* = 4–8 per group. **B** Representative H&E × 10 sections of the main comparator groups, arrow delineates area of severe continued fibrosis in TG animal in vehicle group following bleomycin, not present in other groups. **C** Confirmatory Sircol™ assay data for 12 comparator groups (*n* = 3–10 per group)

TG mice treated with higher dose lanifibranor had significant protection from lung fibrosis compared with those treated with vehicle or lower dose lanifibranor. Although mouse numbers are small, the data suggest a possible dose–effect for lanifibranor in the bleomycin-treated transgenic mice. The mean Ashcroft score in the TG-bleo IVA100 group was 3.82 ± 0.31, compared with that of the TG-bleo vehicle group 5.01 ± 0.38; *P* < 0.01. Treatment with lanifibranor in WT animals did not reduce histological fibrosis (WT-bleo IVA100 3.27 ± 0.44; WT-bleo vehicle 3.25 ± 0.59; not significant). These data suggest that the pro-fibrotic phenotype that develops due to TGF-β upregulation in this model is substantially ameliorated by lanifibranor and is a required environment for lanifibranor efficacy. Figure 3A shows mean Ashcroft score according to each of the 12 treatment groups examined in this study. No significant difference between lanibibranor dose and vehicle was seen in any other treatment group including TG-saline in this study. From these data, administration of bleomycin to a TG mouse resulted in an Ashcroft score on average 2.6 times higher than seen with saline administration in WT mice. This excessive fibrosis was reduced by 24% with the use of lanifibranor at 100 mg/kg. Representative H&E images from the key discriminator groups developed from the Ashcroft scores are shown in Fig. 3B.

To confirm these histological data, we used the Sircol assay as a biochemical measurement of new collagen formation, previously validated as a surrogate of the development of lung fibrosis in mice. New collagen deposition was quantified in each of the 12 subgroups. Figure 3C shows that this technique reproduced the results obtained histologically. There was a dose-dependent significant reduction in collagen deposition in transgenic mice compared to those receiving lanifibranor before bleomycin aspiration. This excessive collagen formation was reduced by 50% with the use of lanifibranor at 100 mg/kg. Notably, there is no significant effect on WT mice after 21 days from oropharyngeal aspiration of bleomycin when compared to saline. This may reflect relatively small number of mice available and greater variability between wild-type samples. Due to the small sample sizes examined, no excess collagen above control was available for WT animals given saline pre-treated with lanifibranor 30 mg/kg. Numerical data are presented below the histogram in Fig. 3C.

### Assessment of pulmonary hypertension development using the pan-PPAR agonist lanifibranor in TβRII∆k-fib mice

To confirm our findings from previous studies on this model, we measured mean arterial blood pressure (MABP) across each of the 6 treatment groups for this experiment. Due to expected procedural difficulties, results for this experiment were available for 33 mice in total. There were no significant differences between treatment groups that may have accounted for differences in right-sided pressures. MABP in these mice was normal.

**Fig. 4:**
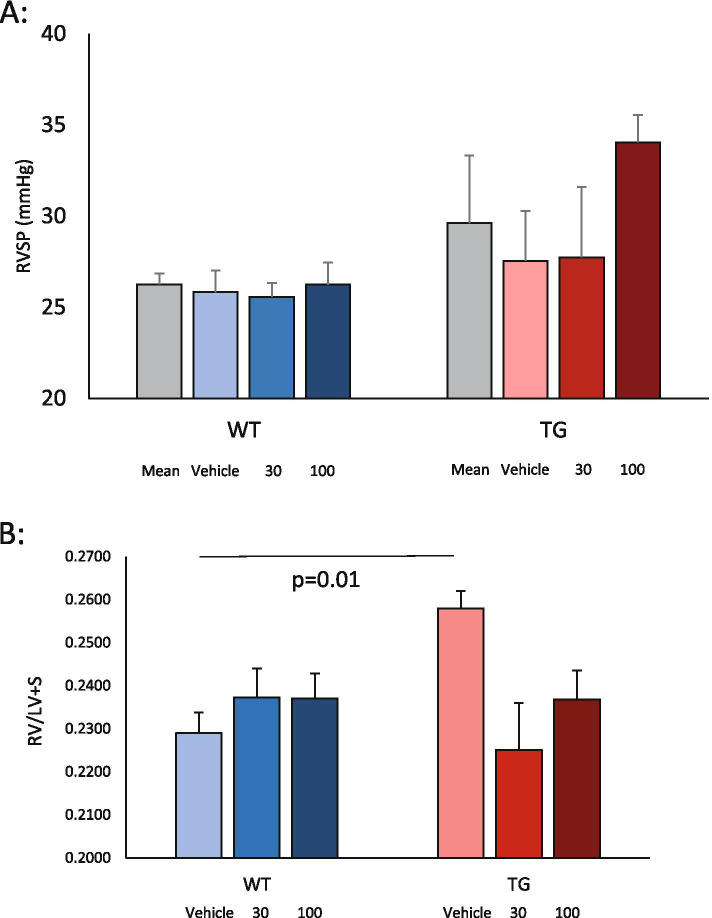
Effects of PPAR agonist lanifibranor in SU5416-induced pulmonary hypertension in TβRII∆k-fib mice. **A** Right ventricular systolic pressure by treatment group (WT/TG with combined mean, vehicle, low-dose and high-dose lanifibranor). **B** Fulton index, RV/LV + S score by treatment group as above

As expected, all mice had slightly elevated right ventricular systolic pressure (RVSP) when compared to published experiments where SU5416 had not been administered. Again, as expected, TG mice developed elevated RVSP compared to WT mice in the vehicle groups with mPAP 25.8 ± 1.2 in WT and 29.0 ± 2.7 in TG. In this experiment, for vehicle-treated mice, this did not reach statistical significance, although numerically is entirely consistent with earlier published studies that reached significance with larger mouse numbers [5, 7] (Fig. 4A). There was no evidence from our results that lanifibranor attenuated the development of pulmonary vasculopathy in this transgenic model since the RVSP was not lower in either the lanifibranor treatment group. In the absence of any attenuation of RVSP elevation, a pooled analysis of the transgenic mouse groups showed pulmonary hypertension compared with wild-type littermate controls (Fig. 4A), in line with the vehicle-treated mice (mPAP for WT 25.4 + 4.0 and TG mPAP 29.7 + 10.6). Consistent with this, histological hallmark lesions of the transgenic mouse model after SU5416 treatment were comparable for lanifibranor- or vehicle-treated mice as described below.

As seen in previous experiments, RV hypertrophy was evident in TG mice in which vehicle was administered when compared to WT littermates (Fig. 4B). Despite no apparent impact on the structural vasculopathy in transgenic mice after SU5416 administration, there appeared to be prevention of right ventricular hypertrophy in the 100 mg/kg lanifibranor treatment group. Thus, compared to vehicle-treated mice, there was no significant increase in RV mass when looking at the treatment groups (for instance, RV/LV + S in TG-SU-100 0.24 ± 0.007; WT-SU-100 0.24 ± 0.005; not significant) and no increase in RV fibrosis seen on picrosirius red stains. This suggests that lanifibranor prevented right ventricular hypertrophy although it is not possible to determine whether this is due to an impact on pulmonary vasculopathy or a direct effect on the ventricle. The latter seems more likely considering that RVSP was not apparently lower in lanifibranor-treated mice in this pulmonary hypertension model.

### Endothelial cell changes in response to lanifibranor in TβRII∆k-fib mice

**Fig. 5:**
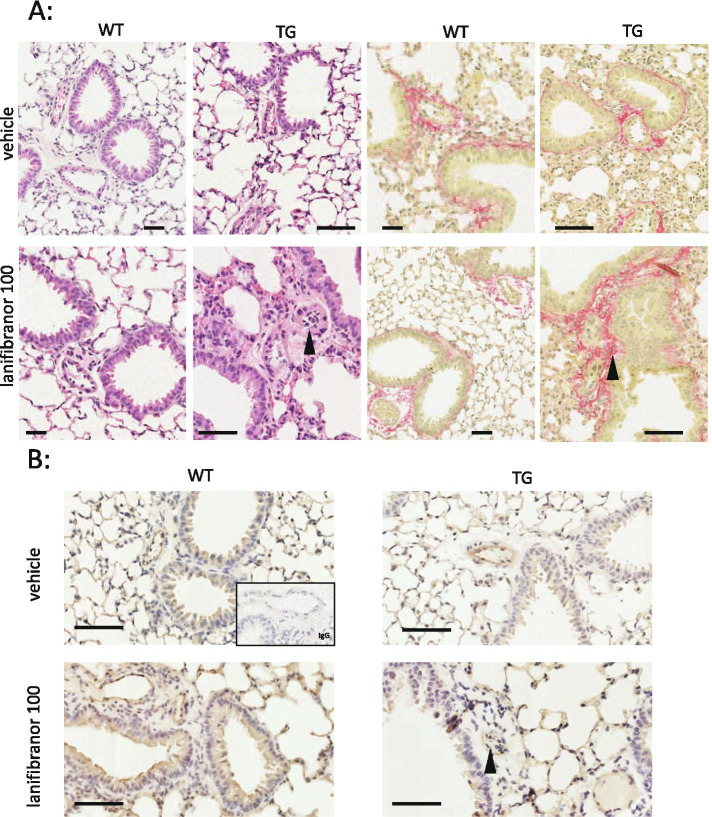
Pulmonary arteriolar structural change following high-dose lanifibranor. **A** Representative H&E and picrosirius red; PSR (× 20) stains for WT and TG vehicle and high-dose lanifibranor-treated groups. Arrows delineate endothelial proliferation in a lanifibranor-treated TG arteriole (H&E) and the presence of excessive perivascular fibrosis (PSR). **B** Representative immunohistochemical stains for CD31 demonstrate endothelial proliferation (arrow) as a cause for elevated RV pressure in this group. Scale bar for each panel is 100 μm

Whilst lanifibrinor treatment may attenuate right ventricular hypertrophy in transgenic mice after SU5416, we did not observe a reduction in pulmonary pressure in this study. Consistent with this, there was no apparent diminution in the endovascular proliferative changes in the SU5416-treated mice between treatment groups. Thus, in this transgenic model, in both vehicle- and lanifibranor-treated animals, we observed luminal obliteration in small and medium-sized pulmonary arterioles, smooth muscle cell hypertrophy and exaggerated perivascular fibrosis (Fig. 5A). Luminal obliterative cells were CD31-positive (Fig. 5B) identifying them as proliferated endothelial cells. This proliferation has been previously described in this model as a result of day 8 endothelial cell apoptosis in response to SU5416, and we therefore conclude that lanifibranor treatment may not alleviate this process.

## Discussion

**Fig. 6:**
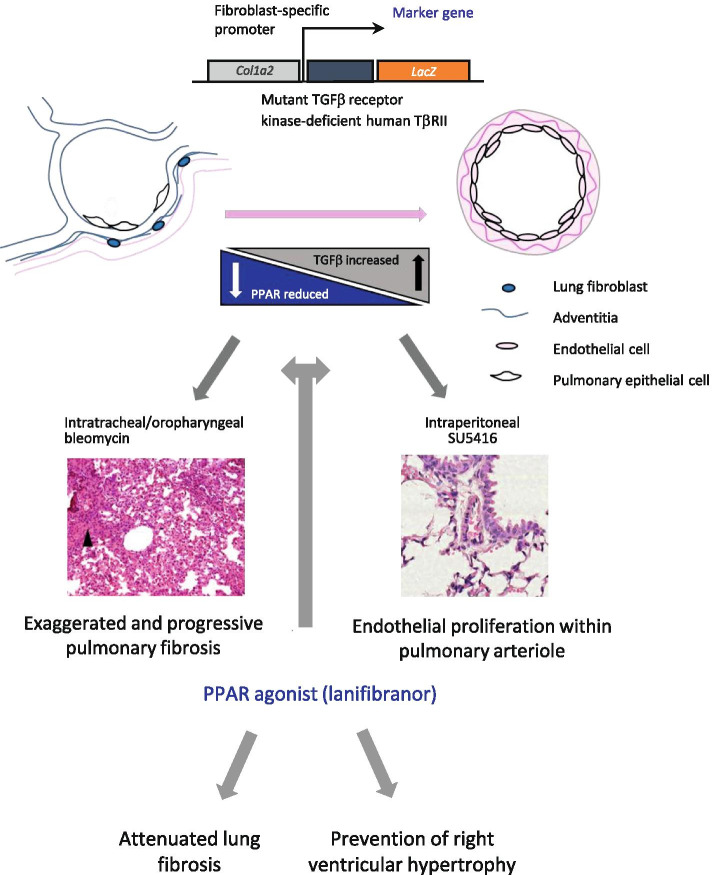
Schematic describing effects of pan-PPAR agonism on pulmonary fibrosis and pulmonary hypertension in TβRII∆k-fib mice. Transgenic mice replicate many features of human systemic sclerosis. The phenotypes of pulmonary fibrosis and pulmonary hypertension are exacerbated by the administration of bleomycin and SU5416, respectively. PPAR agonism alleviates pulmonary fibrosis and prevents development of right ventricular hypertrophy in response to the pulmonary hypertension in this model

Peroxisome proliferator-activated receptors have extensive nuclear effects on many organ systems. In general, agonism of these receptors has been thought to promote cardiovascular health and an extensive literature in pulmonary hypertension suggests overall benefit due to PPARγ activation [13, 19–22]. There is reciprocal repression between TGFβ and PPAR subtypes (with particular focus on PPARγ). In pro-fibrotic states where TGFβ expression is high, PPAR expression is therefore downregulated. In this study, we confirm, as expected, the downregulation of all three PPAR subtypes in the TβRII∆k-fib mouse model of scleroderma. This study also confirms previous findings in animal models of scleroderma that PPAR agonism through lanifibranor prevents or ameliorates the induction of lung fibrosis by bleomycin [14]. The findings related to pulmonary hypertension are more complex and are likely to represent the differential effects of PPAR subtype agonism by lanifibranor with particular effect in this animal model because of the TGF-β pathway upregulation. We have demonstrated overall no significant change in pulmonary pressures in this SU5416-induced model in response to lanifibranor administration, but there is significant heterogeneity in this response. This is likely to be a TGFβ specific effect and hence was not seen in previously published animal studies where genetic predisposition to scleroderma-like phenotype was not due to balanced TGFβ upregulation and where a reduction in pulmonary pressure was found in response to lanifibranor [14]. Pulmonary pressure is recognised as difficult to interpret in preclinical models as well as in patients because of its dependence on both right ventricular output and function and pulmonary vascular resistance. Overall, our findings suggest that lanifibranor may attenuate right ventricular hypertrophy or fibrosis in this transgenic model but further studies would be required to confirm this and explore the interplay between the cardiac, vascular and pulmonary phenotype of the mice, just as these factors are relevant in assessment and treatment of systemic sclerosis. The schematic shown in Fig. 6 integrates our findings and is consistent with other recently published work suggesting that the effects of PPARγ agonism are responsible for the protection of cardiac myocytes through maintenance of mitochondrial structure and function and epigenetic mechanisms involving repression of genes promoting fatty acid oxidation (Cpt1b and Fabp4) [13, 23]. It is notable that these families of genes were shown to be downregulated in our model (Fig. 1A). In human pulmonary hypertension, right ventricular failure is the key predictor of morbidity and mortality, and hence, this finding warrants consideration [24] since the improvement in right ventricular function may have beneficial effects despite no impact on the endothelial proliferative lesions underlying our SU5416 induced transgenic mouse model of systemic sclerosis-associated PAH.

The PPAR pathway genes identified as downregulated within the lung and vascular tissue in transgenic mice are mainly involved in fatty acid degradation (including through oxidation), which is generally considered a positive cellular function to produce energy [13]. Our results are not indicative of highly dysregulated PPAR pathway expression in this mouse model and whilst several clinical disease states have been described related to a mutation in these genes, we confirm that transgenic mice do not develop a similar phenotype. However, as has been seen previously in this animal model, stressors to epithelial and endothelial cell function have resulted in exaggerated effects, including pulmonary hypertension in response to SU5416.

This complex and tissue-specific agonism to each PPAR subtype would predict significant beneficial effects in a disease like systemic sclerosis. Unfortunately, the phase II proof-of-concept clinical trial reported in 2019 did not reach the primary endpoint and further development in this area was discontinued (http://inventivapharma.com/2019/02/results-from-phase-iib-clinical-trial-with-lanifibranor-in-systemic-sclerosis/). This result follows similar studies investigating pharmacological products with PPAR-related effects including imatinib [18] and reflects the complex and microenvironment-specific effects of PPAR subtype agonists and antagonists and the difficulty in balancing effects at the cellular level.

This study used a genetic model which has been characterised previously both phenotypically and at the cellular level for both pulmonary fibrosis and pulmonary hypertension development [7]. In line with previous experiments on this model, we performed these experiments using vehicle and two-dose groups, with the minimal effect seen in the lower dose group supporting previous studies by different groups. However, we did not demonstrate reduced fibrosis in wildtype littermate mice which may reflect different mouse strain genetic backgrounds for our littermate mice compared with inbred wild-type mice used in these previous studies [14]. Unlike previous models, this strain has a well-characterised TGF-β-dependent phenotype and requires a second triggering agent (either bleomycin or SU54216 injection) that allows us to characterise both prevention and treatment effects of pharmacological agents. This study assesses the preventative model.

There are always limitations of translating pre-clinical genetic models to a pathophysiologically complex and longstanding human disease, and difficulties are well-described in systemic sclerosis research. Unfortunately, further trials of lanifibranor in systemic sclerosis are unlikely due to a negative primary outcome for skin disease in a well-designed phase II clinical trial. However, lanifibranor is a promising potential therapeutic option in other fibrotic diseases most notably non-alcoholic steatohepatitis where a successful phase IIb clinical trial has recently been reported with a significant reduction of liver fibrosis. This clinical benefit is supported by a positive impact on portal progression and fibrosis in a highly relevant model of rat cirrhosis [25].

## Conclusions

We have investigated the effect of lanifibranor in a transgenic mouse model of systemic sclerosis. We confirmed TGF-beta-induced effects on PPAR expression in this model and attenuation of the severe and persistent bleomycin-induced pulmonary fibrosis that is a hallmark of the model. We also found that transgenic mice treated with lanifibranor were protected from developing significant right ventricular hypertrophy secondary to pharmacologically induced pulmonary hypertension in this model, although the underlying pulmonary vasculopathy in this specific mouse model was not prevented by lanifibranor.

## Data Availability

The datasets used and/or analysed during the current study are available from the corresponding author on reasonable request.

## Abbreviations

GSEA: Gene set enrichment analysis
H&E: Haematoxylin and eosin
HPF: High-power field
MABP: Mean arterial blood pressure
NO: Nitric oxide
PPAR: Peroxisome proliferator-activated receptor
PSR: Picrosirius red
RNA: Ribodeoxynucleic acid
RVSP: Right ventricular systolic pressure
SMA: Smooth muscle actin
SMC: Smooth muscle cell
SSc: Systemic sclerosis; scleroderma
TG: Transgenic
TGFβ: Transforming growth factor beta
WT: Wildtype
